# Paradoxical Worsening of Chronic Spontaneous Urticaria Following Omalizumab Administration: The Missing Link

**DOI:** 10.7759/cureus.61453

**Published:** 2024-05-31

**Authors:** George N Konstantinou, Indrashis Podder

**Affiliations:** 1 Department of Allergy and Clinical Immunology, 424 General Military Training Hospital, Thessaloniki, GRC; 2 Department of Dermatology, College of Medicine and Sagore Dutta Hospital, Kolkata, IND

**Keywords:** omalizumab, toxocara canis, echinococcus granulosus, parasitosis, urticaria

## Abstract

Omalizumab, a humanized anti-IgE monoclonal antibody, is commonly employed in the treatment of antihistamine-refractory chronic spontaneous urticaria (CSU), where it significantly reduces free IgE levels, minimizing histamine release from basophils and mast cells. Despite its efficacy, there are concerns regarding its effect on parasitic defense due to IgE's role in combating parasitic infestations. We present a case of a 28-year-old female agriculturist with a six-month history of CSU who experienced a paradoxical exacerbation of her symptoms following an increase in the omalizumab treatment dose. This deterioration coincided with a serologically confirmed parasitic infection with *Echinococcus granulosus* and *Toxocara canis*. Despite normal eosinophil counts and IgE levels, which are typically used to identify parasitic infections, the patient's clinical worsening prompted further investigation that led to the identification of the parasitic infection. Treatment with albendazole and omalizumab discontinuation led to the resolution of her CSU, suggesting that the parasitic infection was contributing to the symptom exacerbation. This case highlights the need for careful screening for parasitic infections before initiating omalizumab in antihistamine-refractory CSU patients from endemic regions, or patients who deteriorate clinically on omalizumab, especially when other indicators such as eosinophil count and IgE levels might not suggest infection. It also underscores the importance of considering a tailored approach to managing CSU that balances effective treatment with the potential for adverse effects related to immunomodulation.

## Introduction

Omalizumab, a humanized anti-IgE monoclonal antibody, is approved for treating antihistamine-refractory chronic spontaneous urticaria (CSU) and is positioned as the second step in the international urticaria treatment guidelines [1]. It operates by selectively binding to and lowering free IgE, thus reducing histamine release from basophils and mast cells and ameliorating urticaria symptoms [2]. However, IgE antibodies also play a critical role in defense against parasitic infestations, suggesting that omalizumab therapy might inadvertently worsen parasitic conditions by antagonizing IgE [3]. We report an antihistamine-refractory chronic urticaria patient whose disease worsened following omalizumab therapy.

## Case presentation

A 28-year-old female agriculturist, residing on a farm with four dogs, presented with persistent urticaria without angioedema lasting over six months. Despite the administration of conventional doses of antihistamines, including desloratadine, ebastine, fexofenadine, and rupatadine, no therapeutic response was achieved.

Simultaneously with the onset of urticaria, she reported symptoms of dyspepsia and gastro-oesophageal reflux disease, conditions previously unexperienced. Comprehensive laboratory tests (including complete blood count, liver and renal function tests, and thyroid function tests) were unremarkable. Blood eosinophils were found at 70/μl, and baseline total IgE was 55.5 IU/ml (normal range below 180 IU/ml). Gastroenterological evaluation revealed no evidence of *Helicobacter pylori* infection but indicated mild gastritis, for which famotidine 20 mg orally twice daily was prescribed. Significant improvement in gastrointestinal symptoms was noted three weeks later, yet the urticaria persisted.

During this period, she reported exposure to organophosphates, a group of synthetic pesticides, and was consequently advised to strict avoidance. Subsequently, she altered her environment to eliminate all fertilizers and pesticides for a duration of three weeks. Despite these changes, her condition persisted as resistant to treatment.

Even after the administration of levocetirizine at quadruple the standard dose, her condition did not improve (Urticaria Control Test, UCT: 11, Weekly Urticaria Activity Score UAS7=14. These symptoms, along with the detailed patient history, the physical examination, and the exclusion of other urticarial causes via comprehensive lab tests, met the diagnostic criteria for CSU, characterized by the presence of wheals for more than six weeks without an apparent external trigger. Given the patient's lack of response to typical CSU treatments and her unique environmental exposures, we conducted further investigations to exclude systemic causes. These included evaluations for autoimmune disorders, connective tissue diseases, and an extensive allergy workup, which all returned negative, reinforcing the diagnosis of CSU. Therefore, the addition of omalizumab subcutaneously (SC) at 300 mg every four weeks (baseline total IgE: 55.5 IU/ml) was decided.

After 12 weeks following the first administration, no significant clinical improvement was observed, prompting an increase in the omalizumab dosage to 450 mg Q4 weeks [4] (Table 1).

**Table 1:** Timeline of Clinical Symptoms, Treatments, and Responses *famotidine was prescribed for symptomatology suggestive of mild gastritis CSU: chronic spontaneous urticaria, AE: angioedema, bid: twice daily, qd: once daily, UAS7: weekly urticaria activity score, tb: tablet, OMA: omalizumab

Time Period (after urticaria onset)	Clinical Symptoms	Treatment Administered	Urticaria Outcome/Response
0-3 weeks	CSU without AE	Various antihistamines (tb) (at standard dose) ± famotidine* tb 20 mg bid	No improvement (UAS7=14)
3-6 weeks	CSU without AE	levocetirizine tb 5mg/day (standard dose) up to 20mg/day (quadruple the standard dose)	No improvement (UAS7=14)
6-12 weeks	No improvement in CSU	levocetirizine tb 20mg/day + OMA 300 mg SC	No significant improvement
12-13 weeks	No improvement in CSU	levocetirizine tb 20mg/day + OMA increased to 450 mg	No significant improvement
13-14 weeks	CSU flare and AE	levocetirizine tb 20mg/day + OMA 450 mg + methylprednisolone tb 8 mg qd	Worsening of symptoms (UAS7=35)
14 weeks (parasitosis diagnosis)	CSU and AE ongoing	levocetirizine tb 10mg/day (double the standard dose) + methylprednisolone tb 8 mg qd (discontinuation of OMA)	
14-15 weeks	CSU and AE ongoing	levocetirizine tb 10mg/day + methylprednisolone tb 8 mg qd + albendazole tb 400 mg bid x5 day	Minimal improvement
15-19 weeks	CSU without AE	levocetirizine tb 10mg/day + (methylprednisolone discontinuation)	almost complete CSU control (UAS7≤6)
19-24 weeks	CSU resolution	llevocetirizine tb 10mg/day	Complete resolution of CSU (UAS7=0)
24-27 weeks (last follow-up)	CSU resolution	-	Complete resolution of CSU (UAS7=0)

Unexpectedly, one week after the dosage adjustment, the patient exhibited a deterioration in her CSU (UCT=3, UAS7=35) and reported, for the first time, the occurrence of alternate-day angioedema affecting the lips. Subsequent treatment with methylprednisolone orally (PO) at 8 mg daily yielded little benefit.

At that time, comprehensive blood work and serologic examination, including complete blood count and blood eosinophils, erythrocyte sedimentation rate, liver, renal, and thyroid function tests, anti-TPO and anti-TG antibodies, C3-C4 complement levels, and urine analysis, were again unremarkable. Her serum IgE was 172 IU/ml (normal range below 180 IU/ml), her C-reactive protein (CRP) value was 2.2 mg/L (normal <5 mg/L), and her tryptase levels were 4.1 ng/ml. The stool examination for parasites was negative. Due to the patient’s occupational exposure and despite her normal eosinophil count and total IgE levels, testing for parasitic infection was decided. The serologic examination (semiquantitative ELISA) was positive for *Echinococcus granulosus* and *Toxocara canis* (IgG 2+ and IgM 2+ for both, checked twice). The focus on these particular parasites for testing was informed by their status as endemic to the region in which the patient resided according to the National (Greek) Public Health Organization.

Omalizumab was stopped, levocetirizine PO 10 mg bid continued, and she started albendazole PO 400 mg bid for five days. A follow-up visit after four weeks revealed almost complete CSU control (UCT=16, UAS7≤6), while serologic examination demonstrated IgG 4+ and IgM negative for both of the above parasites. Eight weeks after the last follow-up visit, her disease remained fully controlled (Table 1).

## Discussion

Omalizumab is a highly effective biologic in CSU, with over 70% effectiveness reported by a recent meta-analysis of 67 real-life CSU studies [5]. It was licensed in 2014 for managing antihistamine-refractory CSU. There is robust evidence supporting the use of omalizumab as an add-on therapy for all antihistamine-refractory CSU patients ≥ 12 years old [1].

Omalizumab-induced mild and transient adverse reactions, such as fever, headache, sinusitis, and injection site reactions, have been reported in CSU patients. However, very few reports suggest aggravation of urticaria and/or angioedema following omalizumab administration [6,7]. Ertas et al. [6] documented four patients with antihistamine-resistant CSU who experienced significant adverse reactions following omalizumab therapy. These reactions included urticaria flare-ups, angioedema, and anaphylaxis, occurring at various times after administration. Three patients showed reactions within 12 hours, and the fourth patient exhibited symptoms 12 hours post-administration. Each patient required systemic steroids for management, and the omalizumab treatment was subsequently discontinued in all cases. Ozbagcivan et al. reported an exaggeration of urticaria and angioedema 12 hours after omalizumab administration. The patient required treatment with antihistamines and systemic corticosteroids [7]. The stool examination was negative for parasites. In all these patients, the authors implicated the role of excipients in omalizumab, especially polysorbate, a well-established sensitizer, as the cause of such reactions.

In our patient, a flare-up of urticaria and angioedema of the lips occurred one week after the dosage of omalizumab was increased. This reaction did not happen during the previous three administrations (300 mg subcutaneously), which suggests that hypersensitivity reactions to excipients can be ruled out.

The number of eosinophils and total IgE levels were within the normal range before (55.5 IU/ml) and 14 weeks after omalizumab was started (172 IU/ml). Total serum IgE concentrations after omalizumab administration tend to increase. This occurs due to the formation of omalizumab-IgE complexes, which are cleared from the circulation more slowly than free IgE, in addition to the fact that the larger molecular size of these complexes limits their filtration through the vascular endothelium, leading to their retention within the plasma [8].

Despite normal total IgE levels and eosinophil counts, the decision to test for parasitic infection was based on the patient’s worsening urticaria following a high dose of omalizumab, her agricultural background, close contact with dogs, and the regional prevalence of these parasites. Serological examination revealed evidence of *Echinococcus* and *Toxocara *parasitosis. It remains unclear whether the exacerbation of urticaria could be attributed to a parasitic infection that was present before the initiation of omalizumab, a treatment that may reduce the effectiveness of IgE in parasitic defense, or developed after starting the treatment, provided that omalizumab has been associated with an increase in the incidence of parasitic infections. Indeed, parasitic infestation is a known trigger for CSU aggravation [9]. The European Academy of Allergy and Clinical Immunology (EAACI) guidelines on biological treatments express concerns about omalizumab's impact on the immune system's ability to fight parasitic infections, primarily because it reduces free IgE levels [10]. Furthermore, the European Society of Clinical Microbiology and Infectious Diseases advises considering screening for geohelminths before omalizumab administration in patients from regions where these parasites are endemic [11].

The complex interplay between CSU and potential parasitic infection symptoms should always be taken into account in the differential diagnosis. While the initial symptoms were consistent with CSU, the subsequent unresponsiveness to omalizumab and the presentation of new symptoms (severe angioedema) post-treatment prompted a reevaluation. The subsequent resolution of the patient's symptoms with albendazole treatment, along with the documented seroconversion, not only confirmed the parasitic contribution to her clinical presentation but also highlighted the effectiveness of addressing the root cause of symptom exacerbation. This case illustrates the importance of comprehensive diagnostic evaluations and challenges the assumption that normal eosinophil and IgE levels preclude parasitic infections.

In light of these findings, we advocate for the inclusion of parasitic screening as a routine component of the diagnostic workup in patients with antihistamine-refractory CSU, particularly in regions where parasitic diseases are endemic. This approach ensures that treatment strategies are appropriately tailored, potentially avoiding unnecessary escalation of immunomodulatory therapy and mitigating the risk of worsening underlying infections.

## Conclusions

Omalizumab, a key biologic agent in the management of antihistamine-refractory CSU, has demonstrated significant efficacy in reducing urticaria symptoms in the majority of patients. However, this case highlights a critical consideration: the potential exacerbation of a preexisting parasitic infection or new parasitic infections as a consequence of omalizumab's immunomodulatory effect, particularly in individuals exposed to areas endemic to parasites. Despite normal eosinophil counts and IgE levels, our patient’s CSU symptoms significantly worsened after increasing the omalizumab dose, coinciding with serologically confirmed parasitic infections. This observation underscores the importance of considering a parasitic etiology in patients who deteriorate clinically on omalizumab, especially when other indicators such as eosinophil count and IgE levels might not suggest infection.

This case prompts a reconsideration of the diagnostic and management strategies for CSU, advocating for the inclusion of parasitic screening in the initial workup and ongoing assessment of patients being treated with omalizumab, particularly those from or residing in regions with high endemicity of parasitic diseases. Such measures could prevent unnecessary treatment escalation and avoid potential complications from overlooked parasitic infections. Ultimately, this case highlights the importance of a tailored approach to managing CSU, emphasizing the delicate balance between effective treatment and the avoidance of unintended adverse effects.
